# Evolutionary dynamics of methicillin-resistant *Staphylococcus aureus* within a healthcare system

**DOI:** 10.1186/s13059-015-0643-z

**Published:** 2015-04-23

**Authors:** Li-Yang Hsu, Simon R Harris, Monika A Chlebowicz, Jodi A Lindsay, Tse-Hsien Koh, Prabha Krishnan, Thean-Yen Tan, Pei-Yun Hon, Warren B Grubb, Stephen D Bentley, Julian Parkhill, Sharon J Peacock, Matthew TG Holden

**Affiliations:** National University Health System, 1E Kent Ridge Road, NUHS Tower Block Level 10, Singapore, 119228 Singapore; The Wellcome Trust Sanger Institute, Wellcome Trust Genome Campus, Hinxton, Cambridge, CB10 15A UK; University of Groningen, Hanzeplein 1, PO Box 30001, Groningen, 9700 RB The Netherlands; Institute of Infection and Immunity, St George’s, University of London, Cranmer Terrace, London, SW17 0RE UK; Singapore General Hospital, Outram Road, Singapore, 169608 Singapore; Tan Tock Seng Hospital, 11 Jalan Tan Tock Seng, Singapore, 308433 Singapore; Changi General Hospital, 2 Simei Street 3, Singapore, 529889 Singapore; Curtin University of Technology, GPO Box U1987, Perth, WA 6845 Australia; University of Cambridge, Addenbrooke’s Hospital, Cambridge, CB2 0QQ UK; School of Medicine, University of St Andrews, St Andrews, KY16 9TF UK

## Abstract

**Background:**

In the past decade, several countries have seen gradual replacement of endemic multi-resistant healthcare-associated methicillin-resistant *Staphylococcus aureus* (MRSA) with clones that are more susceptible to antibiotic treatment. One example is Singapore, where MRSA ST239, the dominant clone since molecular profiling of MRSA began in the mid-1980s, has been replaced by ST22 isolates belonging to EMRSA-15, a recently emerged pandemic lineage originating from Europe.

**Results:**

We investigated the population structure of MRSA in Singaporean hospitals spanning three decades, using whole genome sequencing. Applying Bayesian phylogenetic methods we report that prior to the introduction of ST22, the ST239 MRSA population in Singapore originated from multiple introductions from the surrounding region; it was frequently transferred within the healthcare system resulting in a heterogeneous hospital population. Following the introduction of ST22 around the beginning of the millennium, this clone spread rapidly through Singaporean hospitals, supplanting the endemic ST239 population. Coalescent analysis revealed that although the genetic diversity of ST239 initially decreased as ST22 became more dominant, from 2007 onwards the genetic diversity of ST239 began to increase once more, which was not associated with the emergence of a sub-clone of ST239. Comparative genomic analysis of the accessory genome of the extant ST239 population identified that the Arginine Catabolic Mobile Element arose multiple times, thereby introducing genes associated with enhanced skin colonization into this population.

**Conclusions:**

Our results clearly demonstrate that, alongside clinical practice and antibiotic usage, competition between clones also has an important role in driving the evolution of nosocomial pathogen populations.

**Electronic supplementary material:**

The online version of this article (doi:10.1186/s13059-015-0643-z) contains supplementary material, which is available to authorized users.

## Background

The emergence and spread of bacterial pathogens that have become adapted for survival in hospitals poses a major threat to global health systems. One of the most prevalent organisms causing healthcare-associated infections is *Staphylococcus aureus* [1]. This organism is part of the natural microbiota of humans, from which clones of epidemic drug-resistant *S. aureus* have emerged. First described in 1961 within a year of the introduction of methicillin [2], successive epidemic strains of healthcare-associated methicillin-resistant *Staphylococcus aureus* (HA-MRSA) have spread worldwide. Although there is ongoing debate as to whether MRSA is associated with increased mortality compared with its methicillin-susceptible counterpart (HA-MSSA), HA-MRSA infections result in longer hospital stays and higher economic costs, and impact on other resources such as isolation facilities and bed management [3-5].

HA-MRSA was first isolated in hospitals in Singapore in the late 1970s. This was followed in the early 1990s by a sharp increase in prevalence, reaching a plateau in the late 1990s when approximately 40% of *S. aureus* isolated from inpatients with *S. aureus* infections were MRSA [6]. In 2006, a total of 3,517 non-duplicate MRSA isolates were cultured from clinical samples (497 from blood) from the 6 public sector hospitals in Singapore [7]. Several hospitals, including hospitals from this study (Singapore General Hospital and Tan Tock Seng Hospital), initiated MRSA-directed infection control programs from 2006, and the prevalence of MRSA in local hospitals started to drop from 2008 [8].

Prior to 2000, most Singaporean MRSA isolates were multilocus sequence type (MLST) 239 (ST239), which possessed a type III staphylococcal cassette chromosome element carrying the methicillin resistance determinant gene *mecA* (SCC*mec*), and corresponded to the Hungarian pandemic clone [6,9]. Starting in the early 2000s, however, sequence type 22 (ST22) increased in prevalence to become the dominant lineage in Singaporean hospitals. The ST22 isolates belonged to epidemic MRSA (EMRSA)-15, a HA-MRSA clone that emerged in the UK in the mid 1980s [10], and then became endemic in UK hospitals, and subsequently spread internationally throughout Europe and beyond to Australasia. The success of EMRSA-15 is in part due to its inherent resistance to fluoroquinolones associated with an increasing and widespread use of this antibiotic class from the early 1990s onwards, and to its physiological characteristics. *In vitro* studies indicate that EMRSA-15 is a fit clone relative to other HA-MRSA (including ST239), with a higher growth rate and resistance to desiccation [11], although the genetic basis for these characteristics is unknown.

Molecular epidemiology has demonstrated that the global spread of MRSA has primarily been associated with waves of epidemic MRSA clones [12], and that the MRSA populations across geographical regions are in flux over time [13]. Improving our understanding of MRSA population dynamics, and the evolutionary forces that drive this, is crucial for anticipating and combating current and future threats posed by MRSA and other pathogenic bacteria.

Using whole genome sequencing (WGS) we have examined the population structure of MRSA during a period of lineage replacement in Singapore, and investigated the genomes of competing clones for evidence of selection and subsequent evolutionary change.

## Results

### Molecular epidemiology of MRSA in Singapore

**Figure 1:**
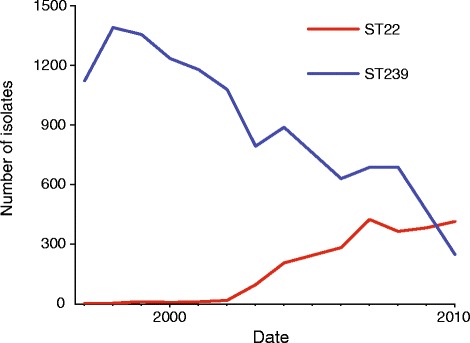
Incidence of MRSA infections in Singapore General Hospital (Hospital 1) from 1997 to 2010 caused by ST22 and ST239.

At the time of the study, Singapore had a population of over 5 million people served by six acute care public sector hospitals. This study focused on MRSA collected between 2000 and 2010 from the three main general hospitals: Singapore General Hospital, Tan Tock Seng Hospital and Changi General Hospital, which will subsequently be referred to as Hospital 1, Hospital 2 and Hospital 3, respectively (Figure S1A in Additional file 1). During this period all of the hospitals experienced a replacement of the MRSA clones associated with clinical infection. Early indications of distinct MRSA populations were based on observed differences in antibiotic resistance profiles; a clone resistant to multiple antibiotics (including trimethoprim-sulfamethoxazole, gentamicin, tetracycline, erythromycin with either inducible or constitutive resistance to clindamycin, and fluoroquinolones) [6] was being replaced by a clone resistant to fewer antibiotics (erythromycin with inducible resistance to clindamycin, and fluoroquinolones). Molecular typing using MLST confirmed the presence of two MRSA clones, and showed that clonal replacement of ST239 by ST22 was occurring. In Hospital 1, the first appearance of ST22 was in 2002, followed by a rise in incidence over the following years such that by 2010 it had become dominant (Figure 1).

MLST and other common molecular typing techniques used to distinguish *S. aureus* lacked the resolution to investigate the fine-scale epidemiological changes occurring in the population. To establish an accurate MRSA population structure in Singapore, we sequenced the whole genomes of 205 randomly selected clinical isolates (Figure S1B in Additional file 1) from 2000 to 2010. Hospitals 2 and 3 randomly selected five to six MRSA isolates each year from a systematic collection of all bacteremia isolates, which was established in 2001 and 2004, respectively. Hospital 1 stored a proportion of MRSA associated with clinical infection each year since 2000, from which isolates were randomly selected from each year. To provide historical genetic context we also sequenced the genomes of all stored and available MRSA isolated in the 1980s (n = 10) and 1990s (n = 28) from Hospital 1 (SGH), the only hospital of the study hospitals to have a historic collection of MRSA for the preceding two decades. To further supplement the historic collection, 17 isolates from a fourth hospital (the National University Hospital; Hospital 4) from 1997 were also sequenced. This fourth hospital was formed in 1985 as an offshoot of Hospital 1, taking with it some of its members of staff and patients.

The 205 isolates from 2000 to 2010 were assigned using sequence data to five MLST sequence types (ST239, ST22, ST78, ST45 and ST5; Figure S1B in Additional file 1; Additional file 2), although the majority were ST239 (n = 110; 54.4%) followed by ST22 (n = 87; 42.6%). Six ST45 strains were isolated in 2009 and 2010 (Additional file 2), and single ST78 and ST5 strains were both isolated in 2004. Analysis of historical isolates from the 1980s and 1990s also revealed a predominance of ST239; all isolates (n = 50) were ST239, with the exception of the very earliest isolates that were ST8 (n = 5). Notably, no other ST8 isolates were identified in the study after 1982. The first ST22 isolates were found within the sample from Hospital 1 in 2003, after which ST22 increased in prevalence and spread to other hospitals (Figure S1B in Additional file 1). Correspondingly, the number of ST22 isolates in our random sample increased from 2003 onwards.

### Phylogenetic reconstruction of the ST239 and ST22 populations

Genome sequence data were used to generate phylogenies based on core single nucleotide polymorphisms (SNPs) for ST239 and ST22. Preliminary phylogenetic analysis revealed that two isolates in the ST22 phylogenies were found on very long branches in comparison to contemporaneous isolates (isolates B11318/05 and DB055850/09). Both of these isolates were obtained from blood cultures, and a review of the clinical records revealed that they were obtained from patients who had been heavily treated with antibiotics for persistent HA-MRSA infection. Neither of the patients had received treatment in an overseas hospital nor had any significant travel history of note, suggesting that greater diversity of the isolates was not likely to be the result of the importation of a genetically divergent strain. Analysis of the genomes of these isolates revealed a deletion of a single guanine residue within the DNA repair gene *radA* for DB055850/09, resulting in a frame-shift mutation, and in isolate B11318/05 a non-synonymous SNP was found in the *mutS* gene encoding a DNA mismatch repair protein. It is likely that these mutations occurred during prolonged antibiotic pressure, resulting in these isolates becoming hyper-mutators. As a result these were excluded from the subsequent temporal analysis.

**Figure 2:**
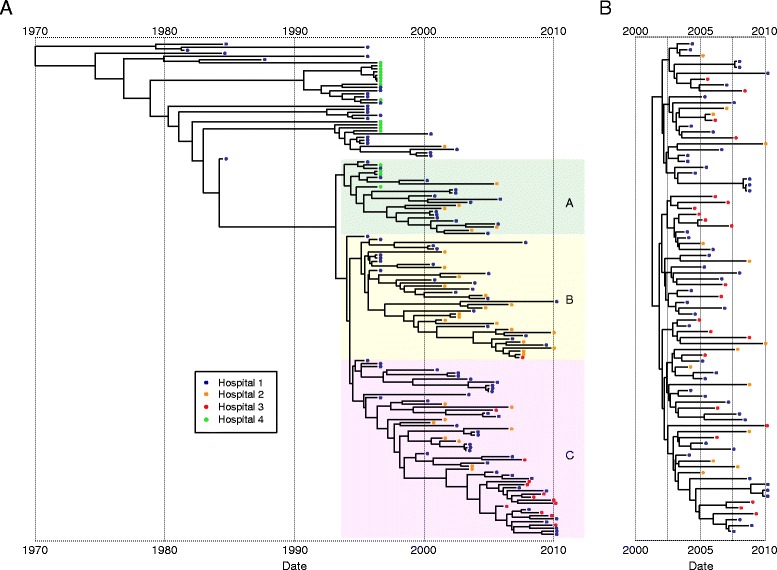
Temporal distribution of Singaporean ST239 and ST22 populations. **(A** **B,)** Maximum clade credibility tree of the ST239 **(A)** and ST22 **(B)** populations based on BEAST analysis. Tips of the tree are constrained by isolation dates; the time scale is shown below the tree. The three colored boxes in the ST239 tree represent the three main ST239 clades present during the study period of 2000 to 2010, clades **A**, **B** and **C**. Isolates that were identified as potential hyper-mutators (Figure 3) were not included in the BEAST analysis. The mutation rate for the ST22 population was calculated as 1.4 × 10^-6^ mutations per year (95% HPD, 1.27 × 10^-6^ to 1.65 × 10^-6^), which was similar to the previously published rate for ST22 [10]. The estimated mutation rate for ST239 of 2.2 × 10^-6^ mutations per year (95% HPD, 1.96 × 10^-6^ to 2.51 × 10^-6^) was slower than the previous estimate of Harris *et al*. [14], but probably reflects the more sophisticated analysis method used in this study and also a more extensive data set. The colored balls on the tips of the tree indicate the origins of the isolate (see legend to figure). For an illustration of the posterior support of the trees see Additional file 3.

Bayesian phylogenetic reconstruction was used to explore the population structure and temporal spread of the two major Singaporean MRSA clones (Figure 2; Additional file 3). Comparison of the two phylogenies showed that the Singaporean ST239 exhibited a greater overall phylogenetic diversity than Singaporean ST22 (Figure 2), consistent with the larger temporal spread of the ST239 isolates. Examination of the ST239 population revealed that the oldest isolates in the collection from the 1980s and 1990s shared a common ancestor dating back to approximately 1964 (Figure 2A; 95% highest posterior density [HPD], 1951 to 1974), around the time of the most recent common ancestor (tMRCA) of the ST239 lineage (tMRCA mid 1960s) [14]. The majority of ST239 isolates from 2000 to 2010 originated from a single lineage that emerged in approximately 1994, and subdivided into three distinct clades shortly after (indicated as clades A, B and C in Figure 2A). In comparison, the ST22 population was predicted to have a tMRCA of July 2001 (Figure 2B; 95% HPD, June 2000 to April 2002), which is contemporaneous with the first epidemiological description of ST22 in Singapore in 2002 [7,15].

During the early to mid-2000s, shortly after the introduction of ST22 into Singapore, the ST239 population was composed of isolates that belonged to the three main sub-clades (colored in Figure 2A) and also some more distantly related isolates, possibly remnants of the early Singaporean ST239 MRSA population, or imports into Singapore from a related population in the surrounding region (Figure 2A). As the ST22 population became increasingly well established, there was a shift in the ST239 population such that by the end our study period, only isolates belonging to two of the three sub-clades were sampled.

In both the ST239 and ST22 phylogenetic trees, isolates from different hospitals were intermingled, indicating that frequent exchanges had occurred between hospitals. This phylogenetic dispersal is consistent with regular inter-hospital transfer of patients and medical staff, which occurred throughout the study period. An exception to this genetic dispersal was noted for ST239 isolates from Hospital 3, which with one exception all belonged to a single sub-clade of the ST239 population (Figure 2A). This clustering contrasted with the ST22 from this hospital, which were distributed throughout the phylogenetic tree (Figure 2B).

### Singaporean isolates in a global context

**Figure 3:**
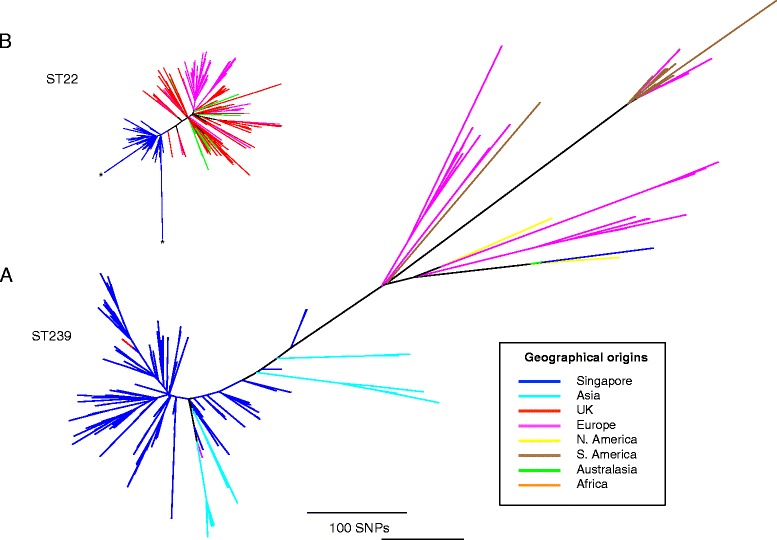
Global context of Singaporean ST22 and ST239 MRSA populations.** (A,** **B)** Unrooted maximum likelihood trees of Singaporean ST239 **(A)** and ST22 **(B)** isolates including additional isolates representative of the global population of these two lineages. The ST239 phylogeny includes the isolates described by Harris *et al*. [14] and Castillo-Ramirez *et al*. [16], and the ST22 phylogeny contains the isolates described by Holden *et al*. [10]. In the case of ST22, only ST22-A isolates were included as these are representative of EMRSA-15, the clone from which the Singaporean ST22 isolates emerged. Trees were built using a maximum likelihood method using SNPs from the core genome, where the ST22 isolate reads were mapped to the reference chromosome of HO 5096 0412 [20], and the ST239 isolate reads mapped to the TW20 [14] reference chromosome. Branches are colored according to their geographical origins (see legend). The scale bar represents substitutions per SNP site for both trees. The two ST22 isolates excluded from the Bayesian analysis as potential hyper-mutators (B11318/05 and DB055850/09) are marked on the figure by asterisks.

We next placed the Singaporean isolates into a global genetic context by expanding both datasets with genomes from global isolate collections sequenced and used previously to reconstruct the origins of both lineages (Figure 3) [10,14,16]. This demonstrated that all of the ST22 isolates from Singapore belonged to EMRSA-15 (Figure 3B), a healthcare-associated MRSA first identified in 1991, and originally defined by phage typing [10]. Recent phylogenomic analysis of a global collection of ST22 revealed that the pandemic clone of EMRSA-15 emerged in the UK in the late 1980s, and quickly spread throughout the UK and beyond [10]. Examining the Singapore isolates in the context of a global collection of ST22 isolates that correspond to the EMRSA-15 pandemic population (referred to as ST22-A2 by Holden *et al*. [10]) showed that they all emanated from a single ancestral node, consistent with the single introduction of ST22 into Singapore, followed by clonal expansion. The isolate immediately ancestral to the Singaporean ST22 clade was isolated in the UK, suggesting a possible origin for this introduction.

By contrast, the Singaporean ST239 population was interspersed with isolates from other regions (Figure 3A). The main clade containing Singaporean isolates also contained two European isolates (UK and Demark) together with other Asian isolates from Thailand and China, suggesting several transmissions to and from Singapore over the 28-year sampling timeframe. One of the European isolates in the Singapore clade was TW20, which was isolated in October 2003 as part of an outbreak on an ICU in a London hospital [17]. A previous study of a global collection of ST239 genomes found that TW20 clustered most closely with isolates from Thailand (85 SNPs to the last common ancestor with a Thai isolate) [14], and it was speculated that TW20 was carried to the UK through intercontinental transmission from Asia. Based on the genetic relatedness of TW20 to the Singaporean isolates (22 SNPs to the last common ancestor with a Singaporean isolate, 4045-41), and the tMRCA of TW20 and the Singaporean isolates of 21st October 2002 (95% HPD 26th April 2001 to 3rd December 2003), it is possible that, within Asia, the origin of this transmission is closer to Singapore than Thailand.

### Competition between MRSA lineages generated selection that has shaped the ST239 population structure

**Figure 4:**
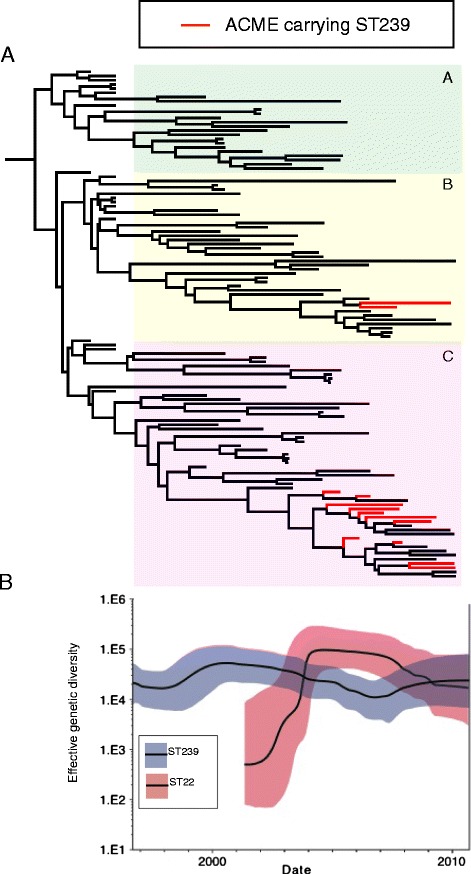
Emergence of ACME-containing ST239 variants in the ST239 population. **(A)** Maximum clade credibility tree of the 2000 to 2010 ST239 populations based on BEAST analysis. Branches containing ACME-containing isolates are colored red. The three colored boxes in the ST239 tree represent the three main ST239 clades. **(B)** Skyline plot representing the effective genetic diversity of the ST239 and ST22 populations over time. The line in the grey shading represents ST239, and the line in the red shading represents ST22, with the shaded areas representing 95% confidence intervals.

The topologies of the phylogenetic trees belonging to the two clones were very distinct. The ST22 tree was composed of branches that radiated out from a central node to produce a star-like phylogeny, whereas the ST239 population had more unbalanced phylogeny. These features suggested that selection has acted differentially on the two populations, and that in the case of ST239 the population had experienced some form of directional selection. Based on the differences in the phylogenies, we hypothesized that competition between ST22 and ST239 for the common niches within hospitals had resulted in selection, which led to differential effects on the two populations. In order to look in more detail at the evolutionary impact, we conducted demographic analysis on the ST239 and ST22 populations (Figure 4). Bayesian skyline analysis [18] provided an estimate of the genetic diversity of the ST239 and ST22 populations. The success of ST22 following its introduction into Singapore was associated with an increase in the genetic diversity of the population from 2002 until 2004, after which this plateaued. During the same period, the genetic diversity of the ST239 population started to decline. Around 2005, when the genetic diversity of ST22 had stabilized, the genetic diversity of ST239 continued to fall until 2007, when, surprisingly, it rebounded and started to increase. At this point the overall clinical prevalence of ST239 in comparison to ST22 in Singapore was falling, and therefore the apparent increase in genetic diversity was likely due to the emergence of successful subpopulations of ST239.

Having observed the signature of an adaptive response in the ST239 population, we conducted comparative genomic analysis to investigate the genetic basis of the apparent success. From the phylogeny it was clear that no single ST239 sub-clone emerged at the time of the increase in diversity (Figure 2); therefore, it was unlikely that mutations in the core genome and clonal expansion were responsible for the observed effect. We then examined the population for evidence of homologous recombination that could be associated with an increase in genetic diversity. This revealed that across the whole ST239 population (isolates from 1988 to 2010) the rate of homoplasy was low; 48 of the 4,349 core SNP sites were homoplasic (1.1%), consistent with previous studies examining ST239 [14]. Analysis of the ST239 chromosomal sequences identified 18 putative recombination regions that corresponded to approximately 7 kb of the chromosome (Additional file 4). However, the distribution of these regions displayed phylogenetic structure, suggesting that, like the SNPs, recombination was not contributing to the increase in genetic diversity.

**Figure 5:**
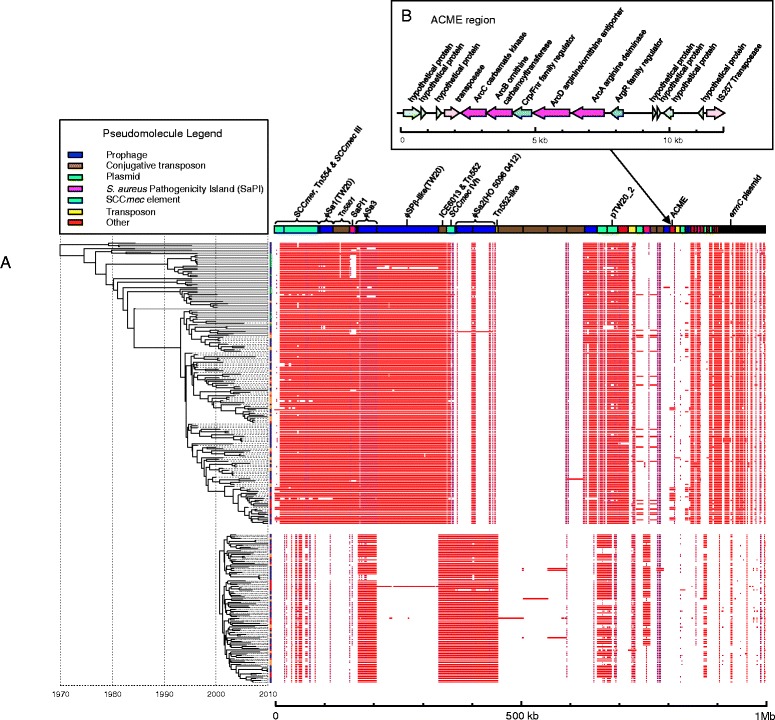
Comparison of the accessory genomes of ST239 and ST22. **(A)** The presence and absence, by mapping, of components of the accessory genomes of the Singaporean ST239 and ST22 isolates is shown in the panel on the right. For the figure, the colors of the blocks represent percentage identity, going from white, indicating absence or less than 90% identity, through to red (via purple), indicating 100% identity. Contigs of the combined accessory genomes are displayed as a pseudomolecule at the top of the panel, and are colored according to the type of MGE (see legend to figure) they belong to, based on contig annotation and also comparison with an MGE reference set. The contigs are ordered from left to right to compile MGEs from the ST239 reference (TW20), and then the ST22 reference (HO 5096 0412) chromosomes (in the order they appear in the chromosome), then additional accessory genome components (in the order of large to small size). Text above the contigs indicates the reference MGE name, or the name of the accessory elements where found. Isolates are ordered according to the maximum likelihood trees displayed on the left; ST239 isolates are displayed at the top, and ST22 isolates at the bottom. Colored balls on the tips of the tree indicate the origins of the isolate (see Figure 2 for legend). **(B)** Gene organization of the type II arginine catabolic mobile element identified in the ST239 pan-genome. Coding sequences, indicated by arrows, are colored according to functional groups: pink, transposition; blue, regulation; magenta, catabolism; green, hypothetical protein.

A possible explanation for the observed increase in the genetic diversity of the population was the emergence of multiple ST239 variants, following the acquisition of mobile genetic elements (MGEs). Analysis of the accessory genome of the ST22 and ST239 populations revealed that, for both lineages, the accessory genomes were relatively stable (Figure 5A); the accessory regions of both the reference genomes (TW20 and HO 5096 0412) were conserved in the majority of isolates. Analysis of the ST22-ST239 pan-genome revealed limited evidence of genetic exchange between ST239 and ST22 populations. In both of these lineages, the prophage component of the genomes appeared to be relatively conserved (Figure 5A; ϕSa1(TW20) and ϕSPβ-like(TW20), and ϕSa2(HO 5096 0412) and ϕSa3(HO 5096 0412), from the ST239 and ST22 reference genomes, respectively); however, we found one of the ST239 isolates (352_06) had acquired a ϕSa2(HO 5096 0412)-like phage, and one of the ST22 isolates (491_05) had acquired a ϕSPβ-like(TW20) phage. This later prophage encodes SasX, a cell wall-anchored surface protein, which is important for colonization and pathogenicity and linked to the success of ST239 in Asia [19]. In the case of the ST22 isolate 491_05, infection by the ϕSPβ-like(TW20) phage has led to lysogenic conversion, introducing the *sasX* into a genetic background where it has previously been unseen. This observation highlights the potential for co-circulating lineages of *S. aureus* to exchange phage and transfer virulence determinants across the population, thereby generating new variants of epidemic clones shaped to the competing population.

The most widely exchanged MGE between the two clones was a small plasmid encoding erythromycin resistance which had been acquired by ST239 isolates. In ST239, erythromycin resistance is typically encoded by an *ermA* Tn*554* transposon associated with the SCC*mer* element [14]; in our collection, 100% of the ST239 isolates contained *ermA*. In contrast, erythromycin resistance in ST22 is frequently mediated by *ermC* encoded on a small 2.4-kb plasmid [10,20]; in our collection, 84% of the ST22 isolates contained *ermC*. Analysis of the accessory genomes of the ST239 isolates revealed that 10 isolates (6%) had augmented their resistome by acquiring the *ermC* plasmid (Figure 5A), to add to the *ermA* they already possessed in their chromosomes.

### Independent evolution of new variants of ST239 carrying the ACME

One of the most abundant novel MGEs in the ST22-ST239 pan-genome (that is, an MGE not present in the reference genomes) was an approximately 12 kb fragment in the ST239 isolates (Figure 5A) that encodes a type II arginine catabolic mobile element (ACME-II; arc^+^ opp-3^-^; Figure 5B) [21]; 14 isolates were identified which contained ACME-II. Comparative genomic analysis of the ST239 isolates from the period when the genetic diversity of the population increased (2006 to 2010), with isolates from earlier in the decade (2000 to 2005), identified that the later isolates were more likely to contain the ACME-II compared with those from the preceding 6 years (28.9% versus 1.5%, respectively). The earliest ST239 strain in the collection that contained an ACME region was isolated from Hospital 1 in 2001 (DB014329/01). This strain was found outside the three main Singaporean ST239 sub-clades, and from the global population context it was most closely related to isolates from Australia and North America.

In contrast, the phylogenetic position of the ACME-positive isolates from 2006 to 2010 indicated that these isolates originated from the Singaporean ST239 population (Figure 4A). Parsimony analysis suggested that there had been a number of potential acquisition events of ACME during this period rather than the expansion of a single clone (Additional file 5). ACME elements were detected in isolates belonging to two of the three ST239 sub-clades. In sub-clade B ACME were present in isolates 097_08 and TTSH1_10, which shared the same phylogenetic node on the tree. Comparative analysis of their ACME regions revealed that the DNA upstream flanking the elements was distinct (Additional file 2). Additionally, we also found that the ACME regions in these two isolates were distinguished by a SNP (in 097_08 there was a SNP in the intragenic region 81 bp upstream of the *arcR* translational start site), suggesting that although the isolates were phylogenetically linked, they may have acquired separate ACME elements. The only other variation in the ACME regions was found in the sub-clade C isolates. The ACME of four of the isolates (CGH4_10, CGH6_06, 158_08 and 006_10) contained a single base deletion in a poly-T octomer 592 bp upstream of the *arcR* translational start. All of these isolates were phylogenetically linked, suggesting a common ancestry for this ACME element variant. The rest of the ACME regions in the sub-clade C isolates were identical and interleaved in the phylogeny with isolates that lacked the element (Figure 4A). From the data it is not possible to identify if the ACME distribution in sub-clade C was the result of multiple acquisitions, or the result of a single acquisition and subsequent loss on multiple occasions.

## Discussion

Singapore has a well-developed and interconnected healthcare system, and is an informative setting for studying the population dynamics of HA-MRSA within a resource-rich healthcare system. Phylogenetic analysis of MRSA isolates from these hospitals over the decade 2000 to 2010 revealed the population structure and dynamics of endemic ST239, and imported ST22, and allowed us to investigate the evolutionary events surrounding clonal replacement. The distribution of isolates from the three hospitals on phylogenetic trees of both ST239 and ST22 is indicative of frequent exchange between hospitals. A recent study in the USA of *S. aureus* from sterile-site infections in patients in four connected hospitals in the Houston metropolitan area demonstrated a similar exchange of isolates [22]. There was some indication of decreased heterogeneity of ST239 in Hospital 3, but this was not evident for ST22 in the same hospital. This may reflect sampling bias since fewer ST239 isolates were available from this hospital compared with ST22, or may be due to differences in the relative referral networks between hospitals. The absence of strong hospital structuring for the MRSA population is consistent with the healthcare model in Singapore, where inter-hospital transfer of patients is common and doctors rotate through different hospitals throughout their training. The results indicate that, in an interlinked hospital healthcare system, MRSA control at one hospital may be compromised due to frequent importation from elsewhere, and requires a unified system of infection control across the different healthcare providers.

The temporal reconstruction of the ST239 population based on the genome data is consistent with the historical epidemiology of ST239 and other MRSA clones in Singapore. Although the numbers were very small, five of six MRSA isolates cultured in 1982 were ST8 (Figure 1B in Additional file 1), suggesting that ST239 may have replaced ST8 in Singaporean hospitals during the early 1980s; however, as the relevant collection no longer exists, it is not possible to elucidate further the very early clone dynamics of MRSA in Singapore.

The work of Holden *et al.* [10], and the data presented in this study, provides strong evidence that the ST22 epidemic in Singapore was the result of a single introduction, possibly from the UK, in 2001. This date is concordant with the first appearance of the antibiotic susceptibility phenotype associated with ST22 in Singapore. Intriguingly our analysis provides evidence that Singapore may have been involved in an intercontinental transmission in the other direction at about the same time. The clustering of the TW20 isolate within the Singapore clade suggests SE Asia as the reservoir of TW20 and the associated London hospital outbreak. Notably the Bayesian estimates of the tMRCA of TW20 and the Singapore population was October 2002. TW20 was originally isolated on the 21st of October 2003, approximately a year after the predicted tMRCA, although the outbreak that TW20 was part of was demonstrated by molecular typing to have started in October 2002. Taken together, the epidemiological and phylogenomic evidence make a convincing case for the direct transmission of TW20 lineage from Singapore to London, leading to the establishment of the outbreak in the hospital shortly after.

A limitation of the sampling framework used as the basis for the study is that the isolates sequenced in this study comprise just approximately 0.5% of all MRSA isolated in Singapore over the study time period. In particular, pre-1999 ST239 isolates were not systematically collected and sampled in the same fashion as the post-1999 ST239 and ST22 isolates. This sampling bias may have generated inexact estimations of the evolutionary paths of both clones. Equally, only isolates from four of six public sector hospitals had been stored and were available to us. However, it is unlikely that the results for the missing hospitals would be significantly different, given similar traffic of patients and healthcare providers and comparable prevalence of MRSA infections [8]. We propose, therefore, that our sampling provided a representative perspective of the national picture.

ST239 and ST22 are two of the most successful hospital-adapted clones of *S. aureus* globally. In Singapore during the study period these clones were in direct competition for the same niche in humans and the hospital environment, which gives rise to questions over the biological basis for succession. There are several plausible explanations for the success of any given nosocomial pathogen. One is antibiotic resistance, but ST22 is more antibiotic-susceptible than ST239, suggesting that resistance to non-β-lactam and non-fluoroquinolone antibiotics was not crucial for its success. The ability to persist in the hospital environment is facilitated by resistance to antiseptics [23]. Notably there was a high level of carriage of antiseptic resistance genes in ST239 (91% of the ST239 isolates contained *qacA/B*), but in contrast, ST22 isolates lacked antiseptic resistance (none of the ST22 isolates contained *qacA/B*; Additional file 2). Rather than resistance, the key to the success of ST22 may reside in the innate physiological properties of this clone. A role for differential growth rate in the success of ST22 is supported by evidence from Knight *et al*. [11], who demonstrated *in vitro* that ST22 (EMRSA-15) isolates had a shorter lag phase and a higher growth rate than ST239 isolates, and also isolates from clonal complex 30 (CC30), which were representatives of EMRSA-16, another successful hospital-adapted epidemic clone. Mirroring the ST239 clone replacement in Singapore, EMRSA-16 emerged in the UK approximately 10 years prior to EMRSA-15 [24], and was the dominant MRSA clone in the UK at the turn of the millennium, before declining in incidence and being replaced by EMRSA-15 [25]. Co-culture experiments by Knight *et al*. [11] demonstrated that ST22 out-competed CC30. Additionally, they showed that ST22 survived desiccation better than CC30, suggesting that it was better adapted to surviving outside the host, thus promoting transmission.

The comparative genomic analysis of the ST239 population performed during this study revealed that new variants of ST239 emerged that had acquired the same mobile cassette of genes, ACME-II, which encodes an arginine-deiminase system (Arc). In the collection, ACME-II was over-represented in ST239 isolates from 2006 onwards. Due to the limited variation in the ACME element, it has not been possible to resolve the precise evolutionary origins and dynamics of the ACME in ST239, but our comparative analysis of the ACME within the ST239 population framework provided strong evidence that the ACME was acquired independently on more than one occasion. These events were predicted to have occurred at a time when the genetic diversity of the ST239 population was declining in the face of competition from the newly established ST22 population (Figure 4).

ACME has been associated with the success of USA300 CA-MRSA [26] and an ST239 clade in Sydney [27]. It has been proposed that the ammonia produced by the ACME-encoded Arc system in the aerobic environment of the skin contributes to acid tolerance during colonization and bacterial persistence [28]. Therefore, it is plausible that acquisition of ACME has enhanced the ability of ST239 to survive on the skin. Consequently, we hypothesize that ACME may provide cells carrying it with competitive advantage, and thus promoted the increased prevalence and persistence of ACME-II-carrying ST239 isolates in Singapore. The most recent epidemiology of MRSA in Singapore would appear to support this; a recent study reported that the proportion of ACME-positive ST239 had risen to an average of 43.7% across all Singapore hospitals [29], suggesting that the population trajectory of the ACME variant of the ST239 we uncovered from the MRSA population-wide genome analysis is robust.

Notably, the type II ACME lacks a spermidine acetyltransferase (*speG*) that is found in the ACME-I associated with USA300 [21,26]. This enzyme acetylates polyamines and has been hypothesized to be a major contributor to ACME-I-mediated fitness in USA300, both to detoxify spermine [28,30] and also in antibiotic resistance, keratinocyte killing, and biofilm formation [31]. Additionally, it has been hypothesized that *speG* has an important role in detoxifying the byproducts of arginine catabolism that are produced by the *arc* system [28]. However, the absence of *speG* suggests that the *arc* operon is sufficient without other components found on other types of ACME to confer an advantage.

The genomic perspective of the MRSA clone replacement in Singapore revealed by this study has highlighted the tenacity of *S. aureus* as a hospital-adapted pathogen. The history of MRSA is one punctuated by the repeated emergence and spread of new clones, supplanting previously successful populations. In Singapore, this has recently been manifested as the displacement of ST239, the dominant MRSA over a number of decades, by ST22, a physiologically fitter MRSA recently emerged from Europe. Arguably these two clones respectively represent the most successful historic and contemporary HA-MRSA pandemic clones of all.

## Conclusions

By examining the MRSA population structure over a decade we have been able to chart the success of ST22 as it established its dominance, and demonstrate knock-on effects on the declining endemic ST239 population, as it is out-competed from its healthcare niche. Our results clearly demonstrate that, alongside clinical practice and antibiotic usage, competition between clones also has an important role in driving the evolution of nosocomial pathogen populations.

## Materials and methods

### Bacterial isolates

MRSA isolates from the period 2000 to 2010 were randomly obtained from the microbiology laboratories of three public sector hospitals in Singapore (Additional file 6), with each hospital contributing a number of isolates each year that approximated to the relative prevalence of MRSA infections among the hospitals. Isolates from Hospital 1 were retrieved from a longstanding HA-MRSA collection (since 2000), whereas isolates from Hospitals 2 and 3 were from blood culture collections that had been in existence since 2001 and 2003, respectively. Pre-2000 Singaporean MRSA isolates were retrieved from the Gram-Positive Bacteria Typing and Research Unit, Curtin University of Technology, Western Australia, where they had previously been deposited in collaborative work with Hospital 1 and Hospital 4 - a university hospital that had been established in 1985, with many existing staff and patients from Hospital 1. In total 260 isolates were sequenced: 160 ST239, 87 ST22, 6 ST45, 5 ST8, 1 ST5 and 1 ST78.

Susceptibility to oxacillin, erythromycin, clindamycin, ciprofloxacin, tetracycline, trimethoprim/sulfamethoxazole, and gentamicin was determined using the Kirby-Bauer disk diffusion method following standards established by the Clinical Laboratory and Standards Institute (CLSI) [32]. The minimum inhibitory concentrations (MICs) of vancomycin were determined via Etest (AB Biodisk, Solna, Sweden), and screening for vancomycin-heteroresistant *S. aureus* (hVISA) was performed on all isolates using Etest GRD strips (AB Biodisk) in duplicate following the manufacturer’s guidelines. DNA was extracted from all isolates using a commercial kit (DNeasy kit; Qiagen) according to specifications required for WGS.

### Tagged genomic library preparation and DNA sequencing

DNA libraries were created using a method adapted from the Illumina Indexing standard protocol. In brief, the steps taken (with clean-up between each step) were: genomic DNA were fragmented by acoustic shearing to enrich for 200 bp fragments using a Covaris E210, end-repaired and A-tailed. Adapter ligation was followed by overlap extension PCR using the Illumina 3 primer set to introduce specific tag sequences between the sequencing and flow-cell binding sites of the Illumina adapter. After quantification by quantitative PCR followed by normalization and pooling, pooled libraries were sequenced on Illumina GAII and HiSeq platforms according to the manufacturer’s protocols generating index tag-end sequences.

### Multilocus sequence type assignment and SNP detection

MLST sequence types were identified from sequence data as previously described [33]. Illumina reads were mapped onto the relevant reference sequences using SMALT [34]; TW20 (accession number FN433596) for ST239 [14], and HO 5096 0412 (accession number HE681097) genome for EMRSA-15 [20]. A minimum of 30× depth of coverage for more than 92% of the reference genomes was achieved for both references (Additional file 2). The default mapping parameters recommended for reads were employed, but with the minimum score required for mapping increased to 30 to make the mapping more conservative. Candidate SNPs were identified using samtools mpileup [35], with SNPs filtered to remove those at sites with a mapping depth less than five reads and a SNP score below 60. SNPs at sites with heterogeneous mappings were filtered out if the SNP is present in less than 75% of reads at that site [14]. Identification of the core genomes was performed as previously described [10,14]. Recombination was detected in the genomes using Gubbins [36].

### Phylogenetic analysis

Phylogenetic trees for ST239 and ST22 were constructed separately using RAxML v.7.0.4 [37] for all sites in the core genomes containing SNPs, using a generalised time reversible (GTR) model with a gamma correction for among-site rate variation [14].

We used the Bayesian software package BEAST (v.1.7.4) [38] to investigate the temporal, spatial and demographic evolution of the ST239 and ST22 populations. To estimate the substitution rates and times for divergences of internal nodes on the tree, a GTR model with a gamma correction for among-site rate variation was used. All combinations of strict, relaxed lognormal, relaxed exponential and random clock models and constant, exponential, expansion, logistic and skyline population models were evaluated. For each, three independent chains were run for 100 million generations, sampling every 10 generations. On completion each model was checked for convergence, both by checking effective sample size (ESS) values were greater than 200 for key parameters, and by checking independent runs had converged on similar results. Models including logistic population models failed to converge so were discarded. Models were compared for their fit to the data using Bayes factors based on the harmonic mean estimator as calculated by the program Tracer v.1.4 from the BEAST package. The best-fit model combination was found to be a relaxed exponential clock model and a skyline population model, and so this combination was used for all further analysis. A burn-in of 10 million states was removed from each of the three independent runs of this model before combining the results from those runs with the logcombiner program from the BEAST package. A maximum clade credibility (MCC) tree was created from the resulting combined trees using the treeAnnotator program, also from the BEAST package. Skyline plots [18] for the ST239 and ST239 groups were constructed using the skyline function of the ape library in R. Ancestral reconstruction of ACME elements was conducted using the Dendropy Python library [39].

### *De novo* assembly and detection of the accessory genome

*De novo* assembly of genomes of all isolates was performed using Velvet v.0.7.03 [40], with mobile genetic elements detected as previously described [14]. In particular, the presence of antibiotic resistance genes and mutations, virulence genes, and genetic elements such as the ACME was screened for by comparison with genetic sequences published on public sequence databases and reviews [41,42].

To create a pan-genome assembly, velvet assemblies for all isolates were mapped against the HO 5096 0412 reference chromosome using the Nucmer algorithm in MUMmer [43]. The core genome for the reference was manually defined by identifying and removing all MGE regions. For each isolate, regions of their assembly greater than 1 kb which did not find a match in the reference chromosome were defined as accessory contigs. These contigs, together with contigs created from the mobile genetic elements identified in the reference genome, were then compared in a pairwise manner using Nucmer. In each comparison, contigs were retained if they contained at least 1 kb of unmatched sequence, or if they were the longer of the two compared sequences. Where two contigs were identical, one was kept at random. All other contigs were discarded. Presence or absence of the resulting accessory genome was assessed for each isolate by mapping their original assembly against the reference core genome regions and accessory contigs simultaneously using Nucmer. Automatic annotation of the accessory genome contigs was carried out using Prokka [44]. Detailed comparisons of individual sequences was conducted on the *de novo* assemblies using BlastN [45], and was facilitated by using the Artemis Comparison Tool (ACT) [46].

### Data access

The Illumina sequences generated and used in this study have been deposited in the European Nucleotide Archive under the study accession number ERP000356.

## Additional files

Additional file 1: Figure S1. Temporal, geographical and clonal distribution of Singaporean MRSA sequenced in this study. **(A)** Map of Singapore indicating the location of the four hospitals contributing isolates to this study. **(B)** Bar graph displays the number of MRSA isolates sequenced over a year, and is broken down according to the hospital of origin. For a given hospital, the columns are subdivided to display the number of isolates of an individual sequence type (ST) that have been sequenced. Additional file 2: Table S1. Isolate metadata, including date and hospital of isolation, mapping and assembly statistics, and genotype and phenotype information. Additional file 3: Figure S2.
**(A,** **B)** Posterior support of maximum clade credibility trees of the ST239 (A) and ST22 (B) populations based on BEAST analysis (as illustrated in Figure 3). Internal branches are colored according to their posterior support (see figure for key). Additional file 4: Figure S3. Prediction of recombination in the ST239 isolate chromosomes. Regions of variation in the core genomes of ST239 isolates predicted to have arisen by homologous recombination in comparison to the TW20 reference chromosome are shown in the panel on the right. Red blocks indicate recombination predicted to have occurred on internal nodes, blue indicates taxa-specific recombination). Isolates are ordered according to the phylogenetic tree displayed on the left. The track along the top of the figure displays the TW20 chromosome and annotation, where protein coding sequences (CDSs) are indicated in light blue. Where recombination is predicted to have occurred in a CDS, the locus tag of the CDS or CDS range affected is displayed in green text above. The associated gene name, where available, is displayed in brackets. Additional file 5: Figure S5. Parsimonious reconstruction of ACME elements in the ST239 population comprising clades A, B and C. The reconstruction of ACME elements was based on the binary presence or absence using a delayed transformation (DELTRAN) method and a maximum likelihood phylogeny. Accelerated transformation (ACCTRAN) was also performed and gave an identical reconstruction. Additional file 6: Table S2. Distribution of HA-MRSA isolates by year and hospital of isolation, and sequence type.

## Abbreviations

ACME: arginine catabolic mobile element
bp: base pair
EMRSA: epidemic methicillin-resistant *Staphylococcus aureus*
HA-MRSA: healthcare-associated methicillin-resistant *Staphylococcus aureus*
HPD: highest posterior density
MGE: mobile genetic element
MLST: multilocus sequence type
MRSA: methicillin-resistant *Staphylococcus aureus*
SCC*mec*: Staphylococcal cassette chromosome element carrying the methicillin resistance determinant gene *mecA*
SNP: single nucleotide polymorphism
ST: sequence type
tMRCA: time of the most recent common ancestor
WGS: whole genome sequencing
