# Supplementary figures and images for: Evolutionary dynamics of methicillin-resistant Staphylococcus aureus within a healthcare system
Source: Genome Biol. 2015 Apr 23;16(1):81. doi: 10.1186/s13059-015-0643-z (PMC4407387; doi:10.1186/s13059-015-0643-z)

Supplemental Fig. S1

(A)

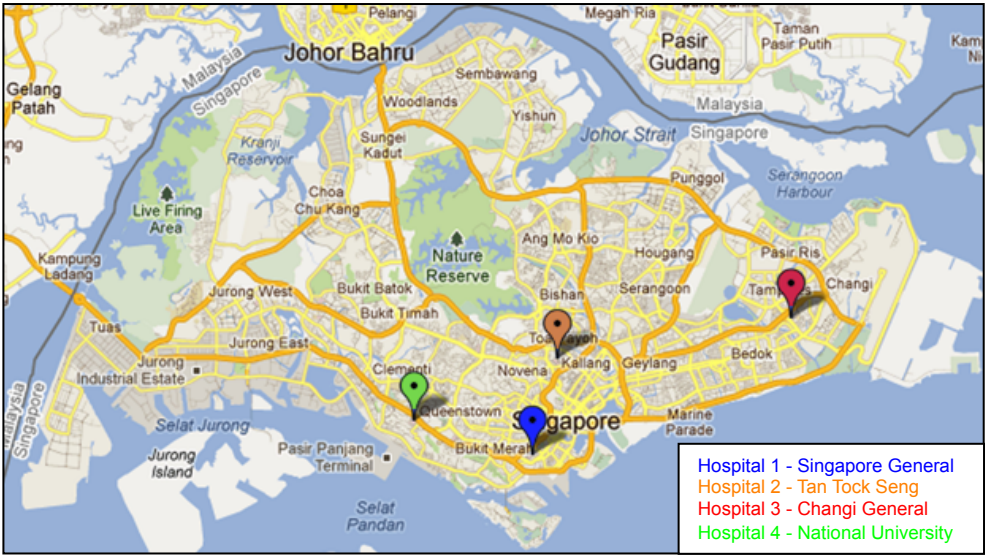

(B)

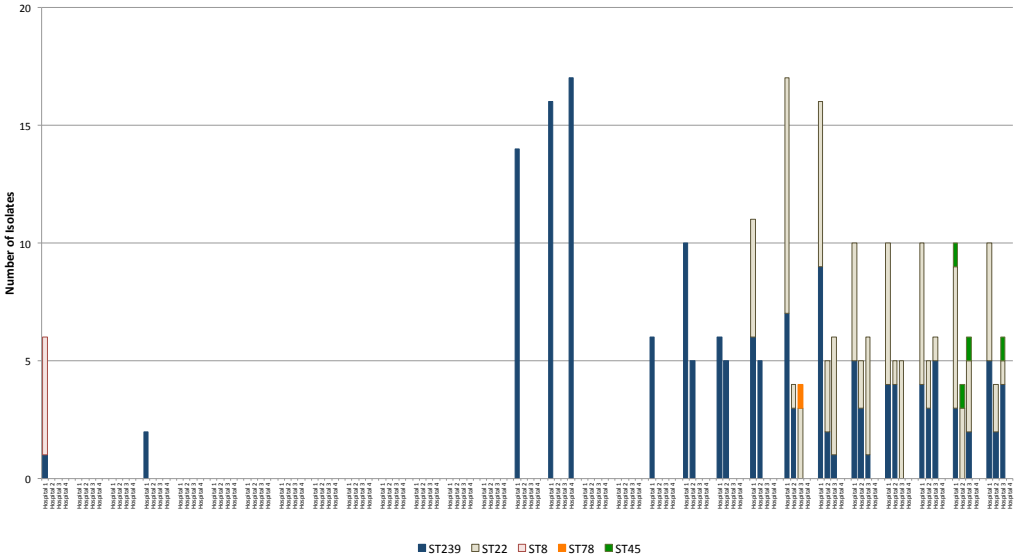

Supplement: Additional file 1: Figure S1. — Temporal, geographical and clonal distribution of Singaporean MRSA sequenced in this study. (A) Map of Singapore indicating the location of the four hospitals contributing isolates to this study. (B) Bar graph displays the number of MRSA isolates sequenced over a year, and is broken down according to the hospital of origin. For a given hospital, the columns are subdivided to display the number of isolates of an individual sequence type (ST) that have been sequenced. [file 13059_2015_643_MOESM1_ESM.pdf]

Figure S2

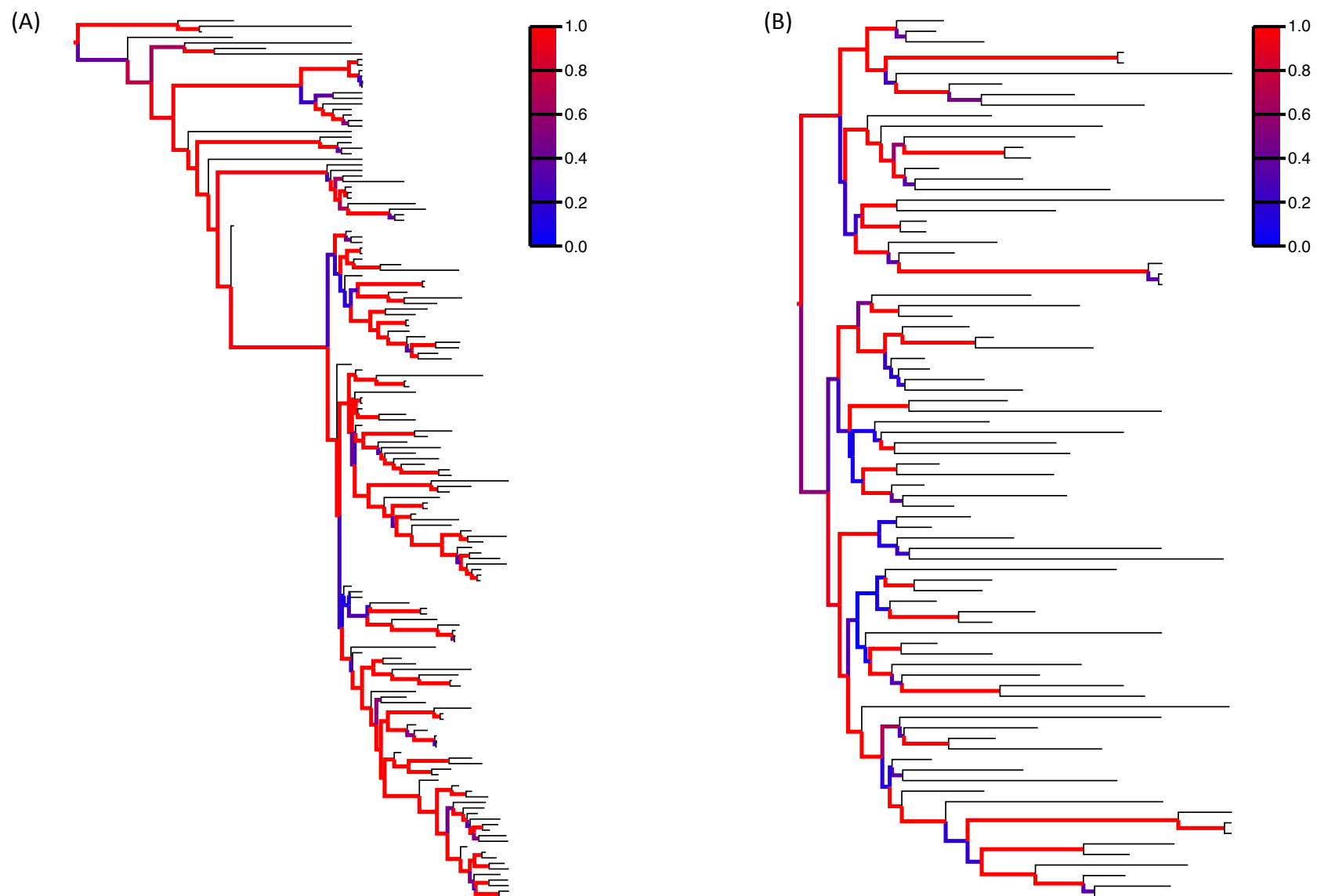

Supplement: Additional file 3: Figure S2. — (A, B) Posterior support of maximum clade credibility trees of the ST239 (A) and ST22 (B) populations based on BEAST analysis (as illustrated in Figure 3). Internal branches are colored according to their posterior support (see figure for key). [file 13059_2015_643_MOESM3_ESM.pdf]

Figure S3

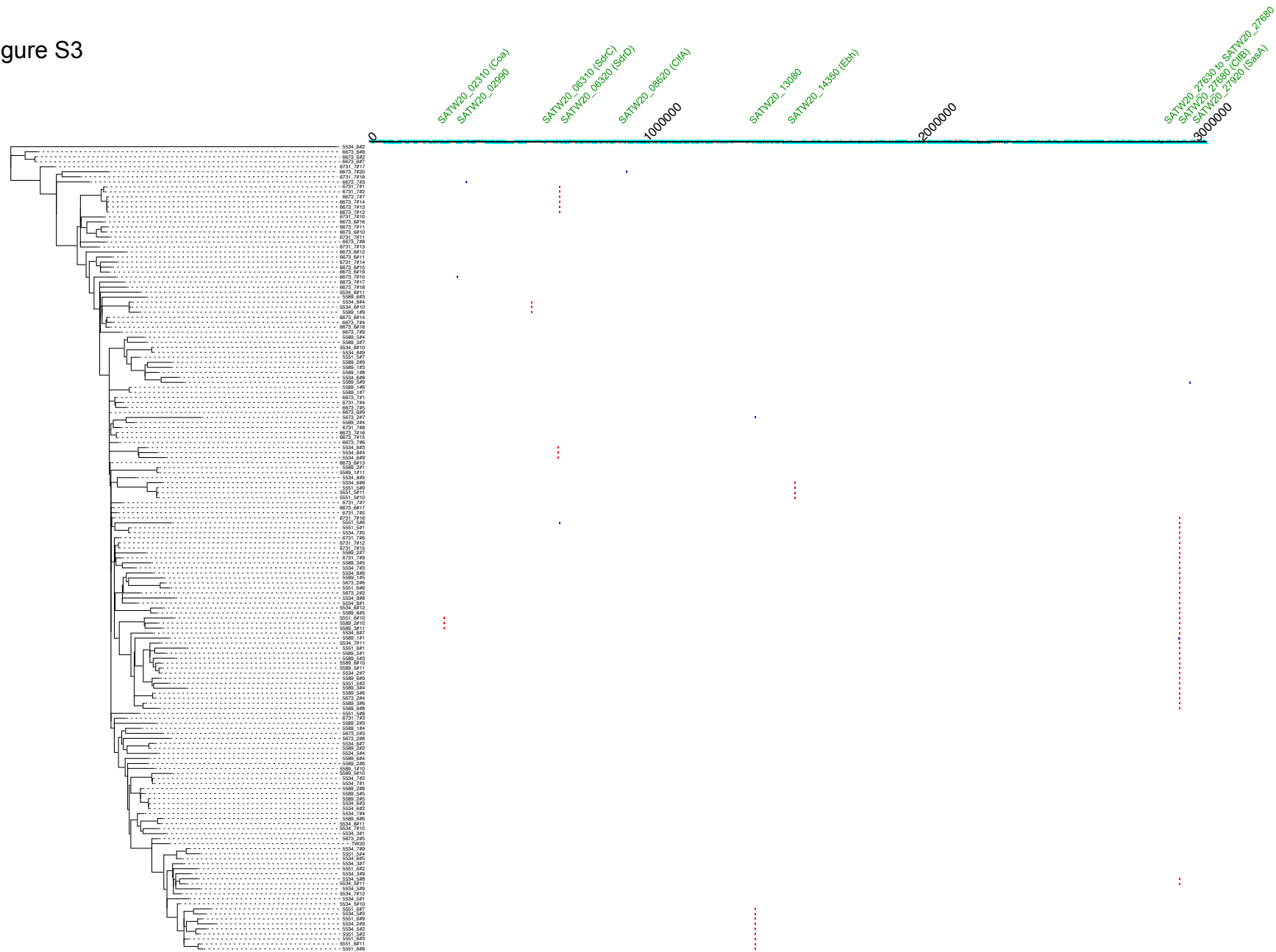

Supplement: Additional file 4: Figure S3. — Prediction of recombination in the ST239 isolate chromosomes. Regions of variation in the core genomes of ST239 isolates predicted to have arisen by homologous recombination in comparison to the TW20 reference chromosome are shown in the panel on the right. Red blocks indicate recombination predicted to have occurred on internal nodes, blue indicates taxa-specific recombination). Isolates are ordered according to the phylogenetic tree displayed on the left. The track along the top of the figure displays the TW20 chromosome and annotation, where protein coding sequences (CDSs) are indicated in light blue. Where recombination is predicted to have occurred in a CDS, the locus tag of the CDS or CDS range affected is displayed in green text above. The associated gene name, where available, is displayed in brackets. [file 13059_2015_643_MOESM4_ESM.pdf]

ACME

Key (ACME): Absent Present

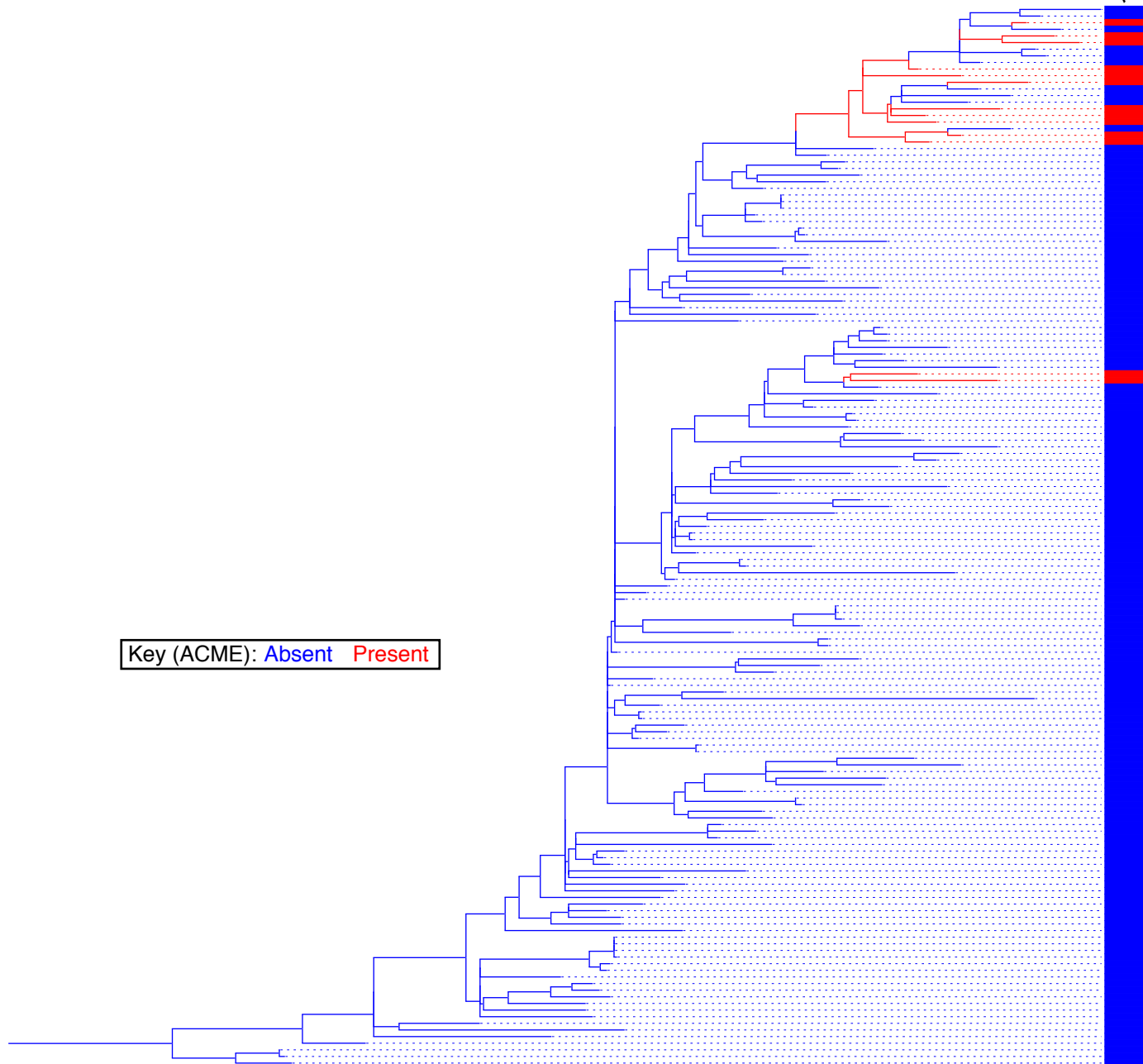

Supplement: Additional file 5: Figure S5. — Parsimonious reconstruction of ACME elements in the ST239 population comprising clades A, B and C. The reconstruction of ACME elements was based on the binary presence or absence using a delayed transformation (DELTRAN) method and a maximum likelihood phylogeny. Accelerated transformation (ACCTRAN) was also performed and gave an identical reconstruction. [file 13059_2015_643_MOESM5_ESM.pdf]
