# Supplementary material for: Evolutionary dynamics of methicillin-resistant Staphylococcus aureus within a healthcare system
Source: Genome Biol. 2015 Apr 23;16(1):81. doi: 10.1186/s13059-015-0643-z (PMC4407387; doi:10.1186/s13059-015-0643-z)
Supplement: Additional file 6: Table S2. — Distribution of HA-MRSA isolates by year and hospital of isolation, and sequence type. [file 13059_2015_643_MOESM6_ESM.pdf]

Supplemental Table S2.

Distribution of HA-MRSA isolates by year and hospital of isolation, and sequence type.

Year	Number of isolates, ST239				Number of isolates, ST22			
	Hospital 1 (Hospital 4)	Hospital 2	Hospital 3	Total (%)	Hospital 1	Hospital 2	Hospital 3	Total (%)
1982*	1 (0)	-	-	1 (16.6)	0	-	-	0 (0)
1985	2 (0)	-	-	2 (100)	0	-	-	0 (0)
1996	14 (0)	-	-	14 (100)	0	-	-	0 (0)
1997**	16 (17)	-	-	33 (100)	0	-	-	0 (0)
2000	6	-	-	6 (100)	0	-	-	0 (0)
2001	10	5	-	15 (100)	0	0	-	0 (0)
2002	6	5	-	11 (100)	0	0	-	0 (0)
2003	6	5	-	11 (68.8)	5	0	-	5 (31.3)
2004***	7	3	0	10 (40.0)	10	1	3	14 (56.0)
2005	9	2	1	12 (44.4)	7	3	5	15 (55.6)
2006	5	3	1	9 (42.9)	5	2	5	12 (57.1)
2007	4	4	0	8 (40.0)	6	1	5	12 (60.0)
2008	4	3	5	12 (57.1)	6	2	1	9 (42.9)
2009****	3	0	2	5 (23.8)	6	3	3	12 (57.1)
2010****	5	2	4	11 (55.0)	5	2	1	8 (40.0)

*Five ST8-MRSA isolates were obtained from Hospital 1 in 1982.

**Seventeen ST239 isolates from Hospital 4 from 1997 were also sequenced. This hospital was formed from the patients and staff of Hospital 1 in 1985.

***One ST78-MRSA isolate was obtained from Hospital 3 in 2004. One ST5 isolate was obtained from Hospital 1 in 2004.

****Four ST45-MRSA isolates (one each from Hospitals 1 and 3; two from Hospital 2) were found in 2009, and one in 2010 from Hospital 3.
